# Increasing isoflurane dose reduces homotopic correlation and functional segregation of brain networks in mice as revealed by resting-state fMRI

**DOI:** 10.1038/s41598-018-28766-3

**Published:** 2018-07-12

**Authors:** Q. Bukhari, A. Schroeter, M. Rudin

**Affiliations:** 1 Institute for Biomedical Engineering, ETH Zurich and University of Zurich, Zurich, Switzerland; 2 Institute of Pharmacology and Toxicology, University of Zurich, Zurich, Switzerland

## Abstract

Effects of anesthetics on brain functional networks are not fully understood. In this work, we investigated functional brain networks derived from resting-state fMRI data obtained under different doses of isoflurane in mice using stationary and dynamic functional connectivity (dFC) analysis. Stationary network analysis using FSL Nets revealed a modular structure of functional networks, which could be segregated into a lateral cortical, an associative cortical network, elements of the prefrontal network, a subcortical network, and a thalamic network. Increasing isoflurane dose led to a loss of functional connectivity between the bilateral cortical regions. In addition, dFC analysis revealed a dominance of dynamic functional states (dFS) exhibiting modular structure in mice anesthetized with a low dose of isoflurane, while at high isoflurane levels dFS showing widespread unstructured correlation displayed highest weights. This indicates that spatial segregation across brain functional networks is lost with increasing dose of the anesthetic drug used. To what extent this indicates a state of deep anesthesia remains to be shown. Combining the results of stationary and dynamic FC analysis indicates that increasing isoflurane levels leads to loss of modular network organization, which includes loss of the strong bilateral interactions between homotopic brain areas.

## Introduction

Characteristics of general anesthesia are loss of sensation, analgesia, loss of muscle control (muscle relaxation), and eventually loss of consciousness. These effects are caused by a temporary change in the brain activity state induced by the anesthetic drug, i.e. changes in local activity patterns and in the activity of functional networks that depend both on the type and the concentration of the anesthetic. How effects on concerted neural activity across the brain translate into the physiological characteristics of anesthesia, e.g. what kind of network changes relate to analgesia or loss of consciousness, is currently not known. EEG has been extensively used for assessing direct neural effects of anesthetic drugs^1–3^, important for guiding their administration. Yet, spatial resolution of EEG recordings is poor. Furthermore it is problematic to extract information regarding the interference of pharmacological interventions including anesthesia with distributed cerebral processing (functional networks).

Analysis of brain function in the absence of a specific stimulation paradigm using functional magnetic resonance imaging (so-called resting-state fMRI, rs-fMRI) has gained tremendous momentum in recent years as it allows identifying functional networks on the basis of the temporal correlation of the signals across brain regions^4,5^. Typically correlation analysis compares signal fluctuations over time intervals of several minutes, which allows identifying ‘stationary’ coherent clusters considered to constitute functional networks. More recently, it has been found that these stationary networks represent temporal integrals of dynamic processes occurring at a much faster time scale. Several approaches have been suggested to study the dynamic aspects of functional connectivity. For example, Leonardi *et al*.^6,7^ have described a dynamic functional connectivity (dFC) algorithm based on a sliding window concept and using a dictionary learning approach. This allowed extracting robust dynamic functional states (dFS), the contributions of which to the overall activity fluctuate as a function of time. The method has been recently transferred to mouse rs-fMRI^8^ revealing dFS exhibits modular structure that reflect interactions within and across major cerebral networks in the mouse. Interestingly this structure was not apparent from the stationary functional connectivity (FC) analysis. Again, the contributions of the dFS to the overall signal were found to fluctuate over time warranting the involvement of many brain areas in information processing. While the value of dFC analysis remains to be shown, it has the potential to reveal insights into network interactions that are not apparent from the analysis of stationary functional connectivity.

Changes in functional network imposed by anesthesia have been investigated both in humans and animals^9–20^. A recent study described dose dependent effects of isoflurane on the stationary cerebral cortical networks in rats^21^. The authors reported that at isoflurane doses higher than 1.5% interhemispheric cortical FC strength was found decreased or completely suppressed, though there was no information to what extent dFSs were affected. The latter point was addressed in a study using macaque monkeys^22^, in which high isoflurane levels were found to decrease the number of dFSs. However, the authors did not investigate the changes in the relative weights of dFS in response to alterations in the level of isoflurane.

There have been two hypothesis linking changes in brain networks to physiological aspects of anesthesia, in particular loss of consciousness: (1) Increase in anesthesia depth decreases functional connectivity within and across brain networks, which will lead to loss of consciousness^10,23^. (2) Increase in anesthesia depth causes synchronization of brain activity across large brain networks. The loss of spatial segregation of information processing then leads to loss of consciousness^24–26^. We addressed these apparently conflicting hypotheses by studying the effects of increasing the dose of the anesthetic isoflurane on the stationary and dynamic FC in the mouse. In particular, we were interested in investigating whether individual stationary and dynamic FC network exhibited differential sensitivity to anesthesia depth, and to what extent the nature of dFS was affected by increasing the isoflurane dose. We carried out the studies in mice considering that the wide range of genetically engineered mouse strains available are well-suited for investigating mechanistic aspects underlying the various physiological characteristics of anesthesia such as analgesia, muscle relaxation, loss of consciousness.

## Results

### Stationary functional connectivity reveals loss of functional connectivity between the bilateral cortical regions with the increasing dose of isoflurane

**Figure 1:**
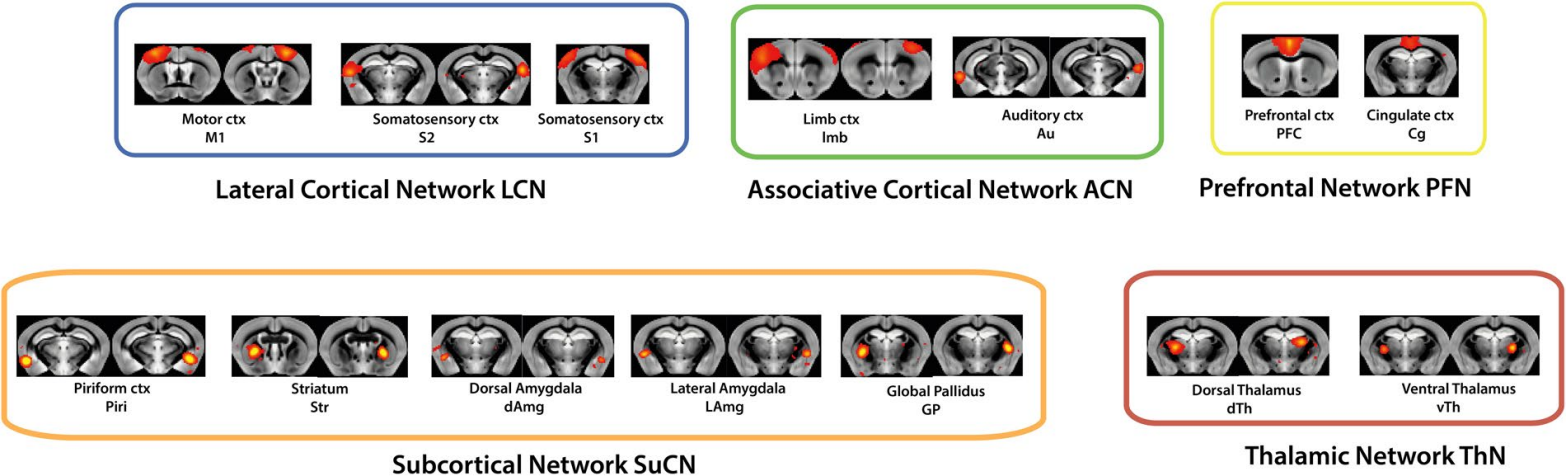
Allocation of independent components (ICs) to functional modules. The 25 ICs identified; have been attributed to the modules defined in^27,28^, i.e. lateral cortical network (LCN), associated cortical network (ACN), prefrontal network (PFN), subcortical network (SuCN) and thalamic network (ThN). Odd lines/columns comprised of ICs located in the left hemisphere and even rows/columns comprised of ICs in the right hemisphere.

**Figure 2:**
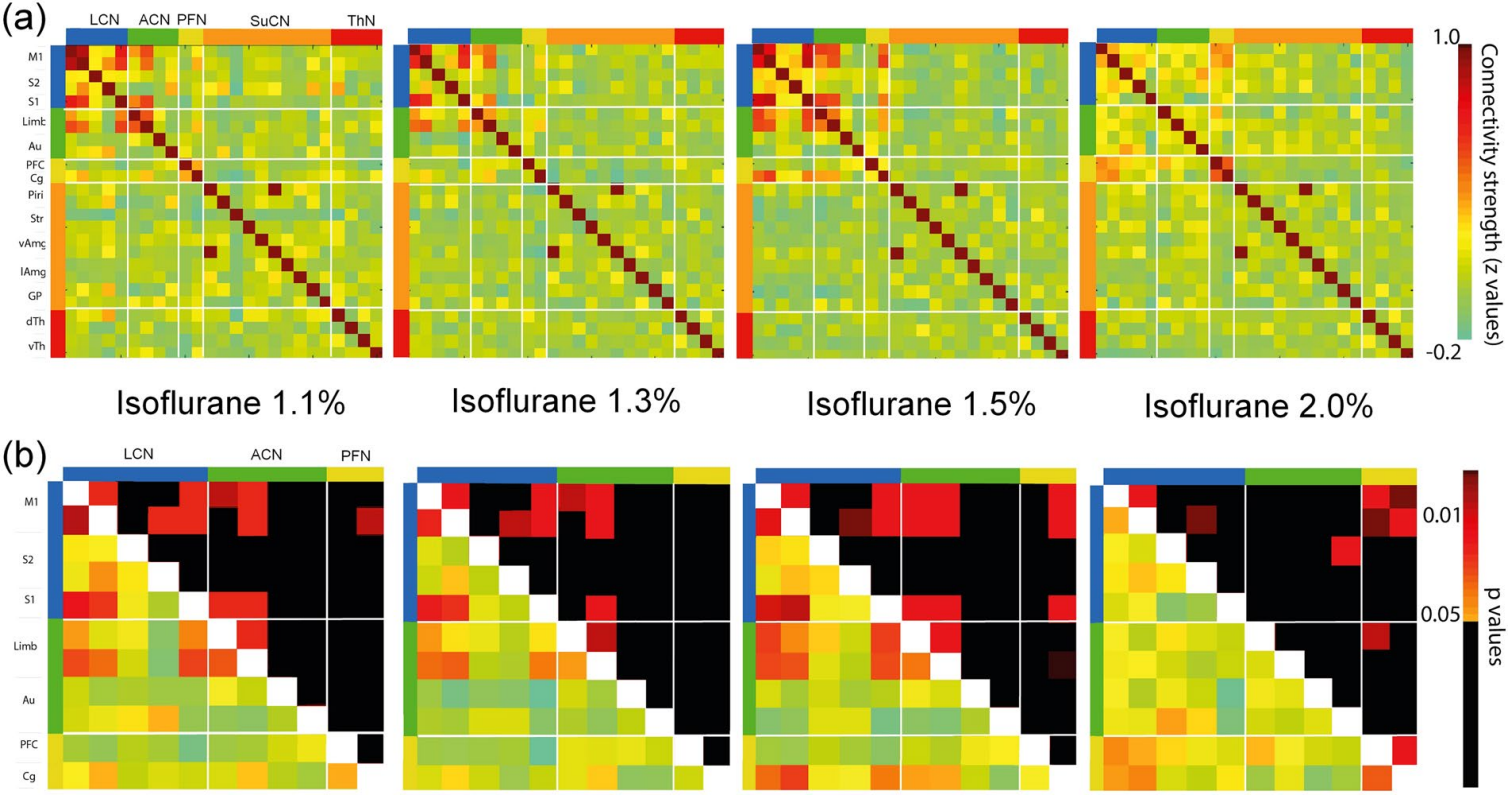
Loss of connectivity between ICs upon increasing isoflurane dose. (**a**) Full correlations between the time series were computed for each isoflurane dose to obtain a stationary functional connectivity matrix. The matrix is organized according to brain networks as defined in^27,28^ with odd rows/columns displaying ICs in left and even rows/columns in right hemisphere. White lines separate individual modules. (**b**) Magnified representation of cortical networks with lower triangle showing z-score and upper triangle showing p-values. Significant within and between network interactions are found for LCN and ACN and to lesser extent for PFN for isoflurane levels between 1.1 and 1.5%. These interactions are largely missing at 2% isoflurane apart from a strong interaction between PFN and M1/LCN. Modules are color coded as (bars on left side and top of correlation matrix): LCN = blue, ACN = green, PFN = yellow, SuCN = orange, thalamus = red. The ICs are labeled at the left. The colour bars indicate the z-transformed correlation values and the p-values, respectively.

Full stationary FC using the full-length time series was computed for each isoflurane dose. The resulting stationary FC matrix is organized according to brain networks as defined in^27,28^. The matrix includes the lateral cortical network (LCN: including somatosensory S1, secondary somatosensory S2 and motor M1 cortex), the associative cortical network (ACN: including limb cortex limb and auditory cortex Au), a prefrontal network comprising elements of the default-mode network (PFN: including prefrontal, PFC, and cingulate cortex, Cg), sub-cortical network (SuCN: including piriform cortex, Piri, striatum, Str, ventral amygdala, vAmg, lateral amygdala, lAmg, and globus pallidus, GP) and the thalamic network (ThN: including dorsal, dTh, and ventral thalamus, vTh). The brain regions corresponding to the individual independent components (ICs) in the matrix are shown in Fig. 1. Significant within-network interactions for LCN and ACN as well as between-network interactions involving LCN-ACN were observed for anesthesia doses between 1.1 and 1.5% isoflurane (Fig. 2). At the 2.0% isoflurane only minimal residual FC have been observed. At all isoflurane levels there was no obvious FC involving subcortical and thalamic network components.

**Figure 3:**
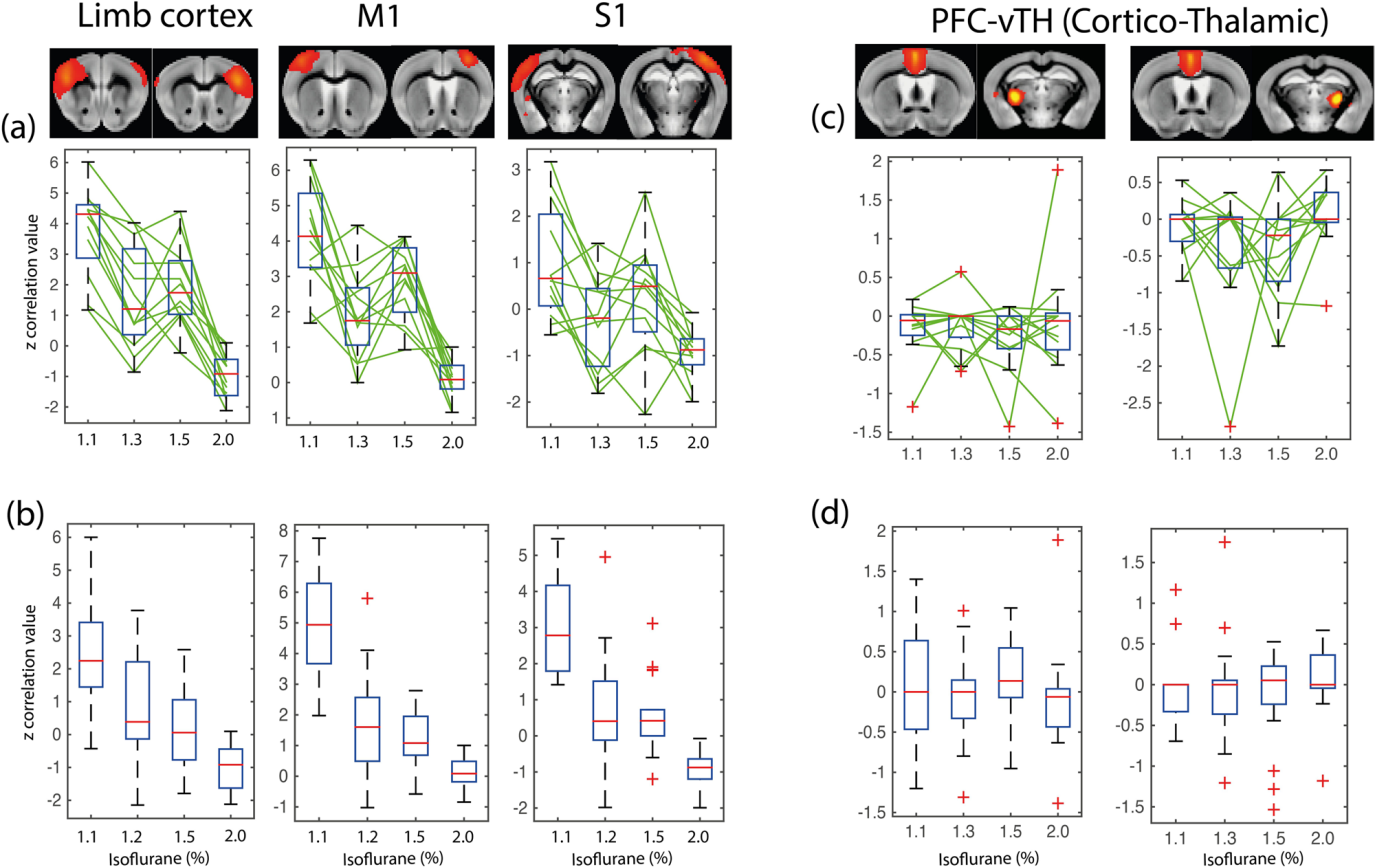
Loss of FC between ICs upon increasing isoflurane dose. (**a**) Loss of FC between bilateral homotopic cortical regions in individual animals upon increasing dose of isoflurane (group 1) for three selected ICs. Overlay images show cortical regions of interest, diagrams changes in z-values, with boxplots indicating mean and the standard deviation (mean±SD) and green lines show changes in individual mice. The animals were kept in the MR scanner as isoflurane dose was increased. The p-values for each comparison are given in Suppl. Table 1. (**b**) Changes z-scores for individuals when exposed to a single dose of isoflurane (group 2). (**c,d**) Absence of FC between prefrontal cortex and thalamus irrespective of isoflurane dose for in individual mice of group 1 (**c**) animal of group 2 (**d**). All results have been corrected statistically using randomized permutations. We applied a t-test separately for every IC component we found. At this stage, it is necessary to correct the resulting p-values for the multiple comparisons across all of the ICs. We have applied a multiple comparison method using permutation testing and build-up a null distribution (5000 random permutations of the subject ordering) of the maximum t-value across all edges. The same unpaired t-test design matrix as for the DR analysis has been used for FDR with multiple corrections. Only ICs found to be significantly different across all groups have been included in the figure.

Dual regression (DR) and network analysis using FSL Nets revealed stationary FC between homotopic regions in the two hemispheres at low isoflurane levels (1.1%). Increasing the isoflurane loss led to a significant reduction in the strength of the homotopic FC as revealed by the respective z-scores (Fig. 2). While this decrease in FC affected all cortical regions, there were quantitative differences depending on the homotopic regions involved. For example, the dynamic range for the decrease in z-transformed partial correlation values of the interhemispheric homotopic connectivity was higher for the somatosensory area representing the limb (Limb) as compared to forelimb motor M1 cortex (M1), which again was higher than in the somatosensory cortex S1. For the highest isoflurane dose, negative z-values have been obtained for Limb and S1, but not for M1. The z-transformed partial correlation values have been corrected using randomized permutations for multiple corrections. No significant cortico-thalamic interaction was identified at any of the isoflurane doses. Even at the lowest isoflurane dose there was no evidence of cortico-thalamic FC (PFC versus vTh; see also Supplementary Material). There was no difference between data obtained from a dose-escalation in individual mice (Fig. 3a) or mice exposed to a single dose of isoflurane (Fig. 3b). In order to identify if there is any dosage effect at all, we applied one-way anova. The null hypothesis assumed no effect of anesthetic dose. The results of the analysis applied to the connectivity between the regions shown in Fig. 1a are given in Supplementary Table 2. The results clearly reveal that the null hypothesis had to be rejected, i.e. the FC between brain regions, that reached the significance level, were found to depend on the dose level of the anesthetic.

### Dynamic functional connectivity analysis reveals loss of spatial segregation across brain functional networks with increasing dose of isoflurane

Dictionary learning was used^6–8,29^ for evaluating dynamic aspects in FC. The atoms or dynamic functional states (dFS) were estimated by concatenating the results obtained from all groups and used as regressor for analyzing rs-fMRI datasets for each individual anesthesia dose similar to the procedure applied in ICA analysis and dual regression. The window length was set to 40-times the repetition time (*TR*), which for a *TR* = 1*s* corresponded to 40 s. The window was advanced across the data set with a step increment of 1 *TR*. The number of atoms were kept at 20. We used the least square projection method of least fitting for the dictionary learning back-fitting. Twenty atoms explaining approximately 50% of the variability were considered for the analysis (Suppl. Figure 1), some of them exhibiting remarkable structure associated to the functional modules (Suppl. Figure 2). For example dFS #1 reflects the interaction of LCN with the SuCN, PFN and ThN as well as PFN with SuCN and ThN, while dFS #9 captures the connectivity between components of the thalamus and cortical and PFN components. Similarly, dFS #10 predominantly reflects FC between LCN/SuCN and the other networks identified including ACN, and dFS #14 captures the interaction of PFN with other networks. The structure of the several dFS revealed distinct interactions within and between the identified networks (Suppl. Figure 2).

**Figure 4:**
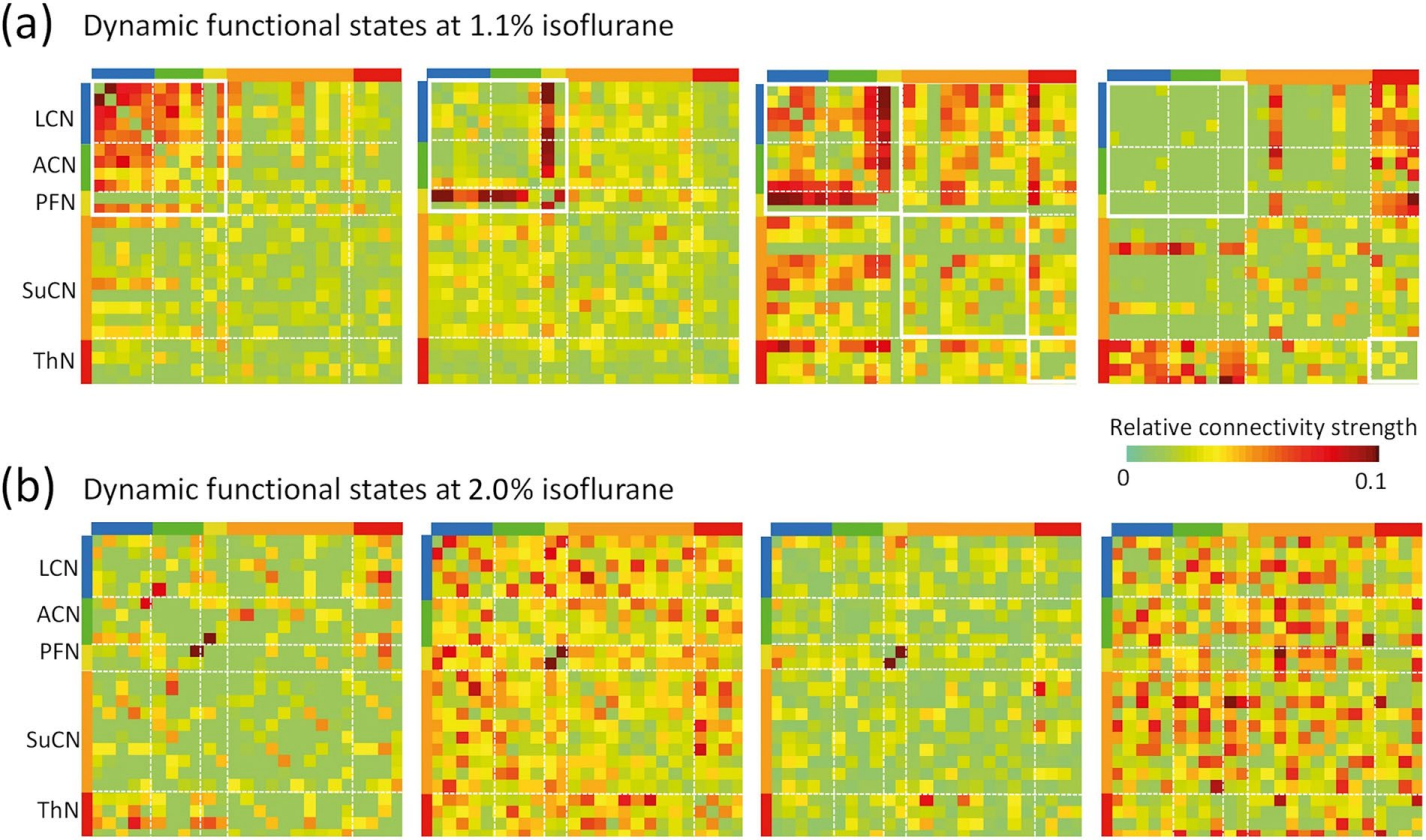
Structure of most relevant dynamic functional states at isoflurane dose of 1.1 (**a**) and 2% (**b**). The figure shows the four dFS obtained for mice anesthetized at 1.1% (a) and 2.0% isoflurane (**b**) associated with the highest weight. White dashed lines separate individual modules, interacting modules a highlighte as white box in upper triangle of correlation matrix. At 1.1% isoflurane dFS with highest weight represent interactions between cortical modules (LCN-ACN; PFN-LCN/ACN) as well as between cortical and subcortical (LCN/ACN/PFN-SuCN; second panel from right) and cortical and thalamic networks (LCN/ACN/PFN-ThN; panel at right). Modules are colour coded as: LCN = blue, ACN = green, PFN = yellow, SuCN = orange, thalamus = red. The colour bar indicates the z-transformed correlation values.

We then applied the dFC algorithm separately to analyse rs-fMRI data obtained for the highest and the lowest isoflurane dose to identify the four dFSs exhibiting the highest weights, i.e. accounting for most of the variability (Fig. 4). While the most relevant dFSs obtained at 1.1% isoflurane exhibited some structure that could be associated with the functional modules (i.e. connecting ACN-ACN and ACN-LCN; ACN/LCN-PFN; ACN/LCN-PFN and ACN/LCN-SuCN; ACN/LCN-SuCN and ACN/LCN-Thal), this was not the case for dFSs obtained at 2.0% isoflurane, which were found to be largely unstructured.

**Figure 5:**
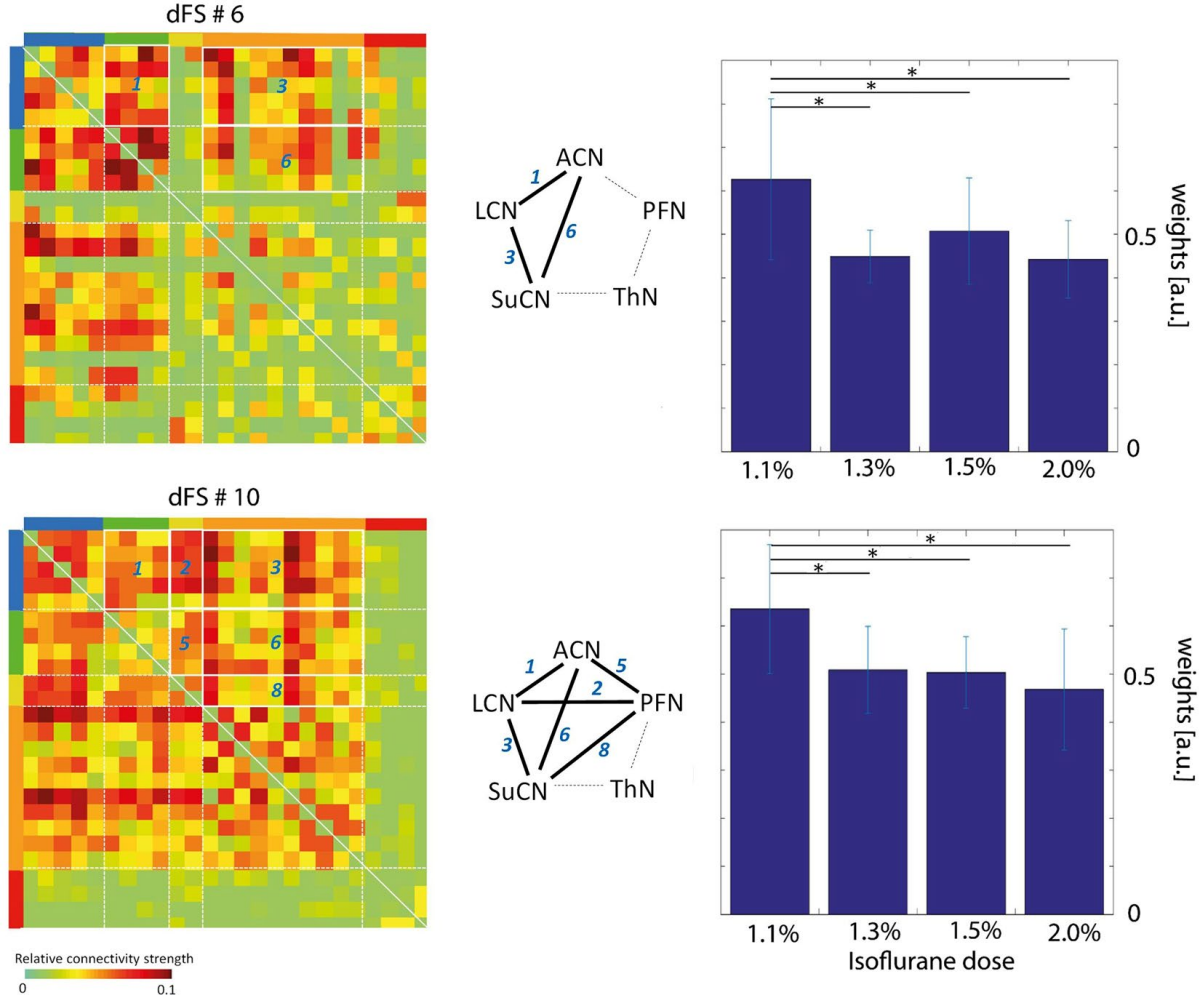
Significant decrease in weight of dFSs as a function of isoflurane dose. Results of dFC analysis for isoflurane doses of 1.1%, 1,3%, 1.5% and 2.0% (group 1). dFS #6 and dFS #10 revealed significant decrease in weight between the lowest isoflurane dose (1.1%) and all other doses tested (FDR corrected, *p < 0.01). These dFSs describe interactions between {dFS #6: LCN-ACN, LCN-SuCN, ACN-SuCN} and {dFS #10: LCN-ACN, LCN-PFN, LCN-SuCN, ACN-PFN, ACN-SuCN, and SuCN-PFN}. Numbers in the correlation matrix and the network graphs indicate interaction between modules. The bar graphs show the mean absolute sum of dFS fluctuations. Asterisks indicate statistical significance.

For analysis of dose-dependent effects, the order of the atoms was kept fixed according to that determined for the lowest isoflurane dose to allow for comparisons across groups. For two of the atoms, we found a significant decrease in weight when comparing results obtained at 1.1% isoflurane with those obtained for all other doses: dFS #6 reflecting the interaction LCN/ACN with SuCN, and dFS #10 capturing interactions between SuCN and the cortical networks (LCN, ACN, PFN). In both cases the weights of the dFS contribution to the overall signal decrease upon increasing the dose of the anesthetic (Fig. 5).

**Figure 6:**
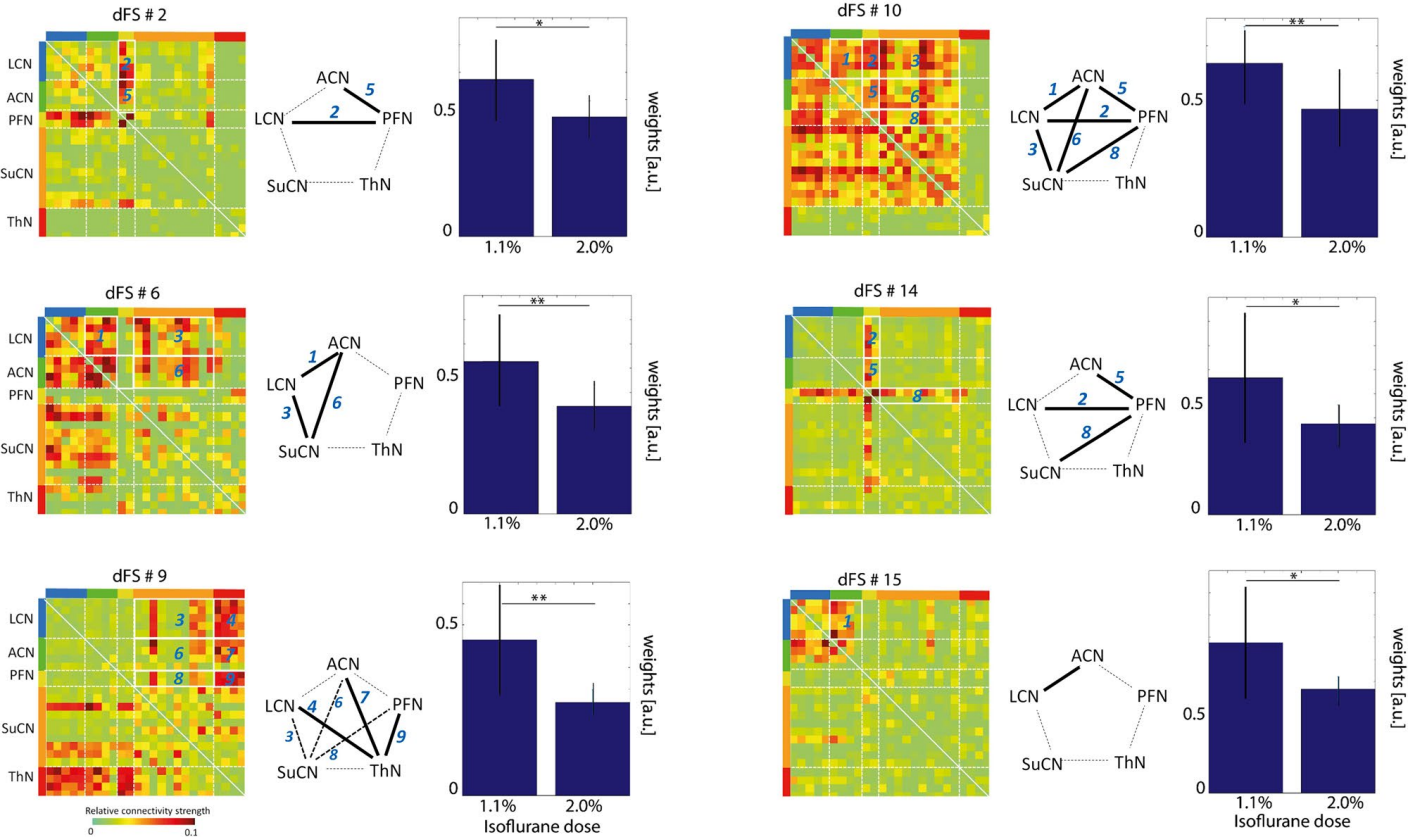
Significant decrease in weight of dFSs comparing lowest and highest isoflurane dose. dFC for isoflurane doses of 1.1% and 2.0% (group 1). dFS #2, dFS #6, dFS #9, dFS #10, dFS #14 and dFS #15 displayed a significant decrease upon increasing the isoflurane dose from 1.1% to 2.0% (*p < 0.01, **p < 0.001, FDR corrected). Numbers in the correlation matrix and the network graphs (center) indicate interactions between modules. The bar graph shows mean absolute sum of dFS fluctuations. dFS #2 and dFS #14 represent the interactions of PFN with other networks, dFS #6 of both LCN and ACN with other networks, dFS #9 of SuCN and ThN with other networks, dFS #10 among all the networks except ThN, and finally dFS #15 within and between LCN and ACN. The 1-p of all the comparisons are shown in Suppl. Table 3.

The weights of additional dFSs exhibited a significant decrease when comparing only groups of mice anesthetized at 1.1% and 2.0% (Fig. 6). dFSs affected describe the interaction between PFN and cortical networks (dFS#2), cortical and subcortical networks (dFS#6), thalamic and cortical networks (dFS#9), cortical and subcortical networks (dFS#10), PFN and cortical/subcortical networks (dFS#14), and LCN-ACN (dFS#15). It should be noted that dynamic FC analysis indicates a significant role for cortico-thalamic FC, which was not apparent from stationary analysis at all (Figs 2 and 3), a finding in line with earlier observations^8^.

### Physiological measurements

**Figure 7:**
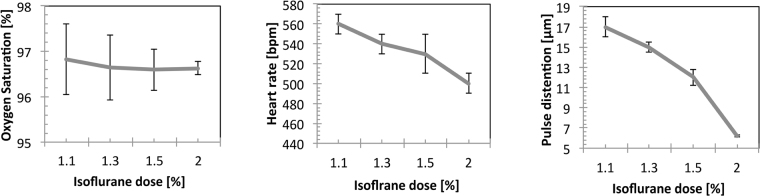
Change in peripheral hemodynamic parameters as a function of isoflurane dose. (**a**) Blood oxygen saturation stayed constant irrespective of isoflurane dose indicating stable respiratory condition in the artificially ventilated mice. (**b**) Heart rate dropped significantly upon increasing the isoflurane dose. (**c**) Similarly, there was a substantial decrease in pulse distention as the anesthesia level was increased. All values are given as mean ± SD.

Anesthesia is known to have profound effects on respiration and cardiovascular output. As animals were mechanically ventilated, respiratory depression upon increasing the anesthesia level could be avoided as reflected by the stability of the blood oxygen saturation (Fig. 7a). Nevertheless, there was a dose dependent effect on the cardiovascular parameters heart rate (Fig. 7b) and pulse distention (Fig. 7c), a measure of vessel pulsatility, and thus related to blood pressure. Both parameters significantly decreased as a function of the isoflurane dose.

In order to identify potential effects of alterations in systemic physiological parameters on functional connectivity and dynamic functional states, we carried out a statistical analysis testing for significant group differences using heart rate or pulse distention values as correlators. We did not find any significant effects on FC (Supplementary Material).

## Discussion

Analysis of stationary FC, i.e. FC patterns integrated over time intervals of several minutes, in humans, monkeys, and rodents, has revealed significant anesthesia-induced alterations when compared to the awake state^24,25,30–32^. Nevertheless, this ‘static’ network view will be inherently incomplete as brain activity states are known to fluctuate at a much shorter time scale^33,34^ and the characterization of these dynamic functional states may constitute an important aspect for understanding anesthesia effects.

There are two major reasons for studying effects of anesthesia in mice. Firstly, the large majority of fMRI studies in rodents including mice use anesthesia in order to minimize confounding contributions due to physiological stress and motion. This leads to biased activity patterns as anesthesia inherently interferes with brain function as evident by anesthetic-specific effects on brain networks^14,35,36^. The effect on brain activity states does not just depend on the nature of the anesthetic drug, and hence its specific mechanism of action, but also on its dose, which determines anesthesia depth. Understanding these influences is essential for proper interpretation of fMRI results. Secondly, the possibility to modulate anesthesia effects on functional brain states by targeted pharmacological or genetic interventions may reveal mechanistic aspects on the anesthetics mode of action.

Isoflurane has emerged as attractive anesthetic for fMRI studies as it allows maintaining stable anesthesia condition suitable for functional imaging studies in rodents^24,37,38^ and non-human primates^25,39^. Despite its widespread use, the mechanisms by which isoflurane induces anesthesia, are poorly understood. Obviously the drug’s action is not specific as it has been reported to interfere with GABA, glutamate, and glycine receptors^40,41^. It is well documented that isoflurane at higher doses leads to increases in cerebral blood flow thereby affecting neurovascular coupling as well as the hemodynamic baseline state, which will modulate fMRI readouts^42^. Also, its interference with the GABA neurotransmitter system may be limiting when studying GABAergic processing. Anesthetics such as medetomidine, an α-adrenergic agent, have been suggested as alternative^43^; however their suitability is subject to other limitations. Medetomidine is vasoconstrictive and may lead to side effects such as convulsion. Combined use of medetomidine and isoflurane at low dose allows controlling confounding side effects to a large extent and moreover allows studying cortical and subcortical networks^14^. Though combination anesthesia is increasingly used in functional imaging studies, it is important to understand the effects of each compound individually in order to analyze potential synergistic or antagonistic interactions. We therefore focused in the current work on characterizing effects of isoflurane on stationary and dynamic functional states in relation to its dose.

There are two hypotheses regarding anesthesia effects on brain function. The first hypothesis suggests anesthesia disrupts functional networks of the brain such that individual brain regions become increasingly disconnected. Decreased functional connectivity within and across brain networks would impair information processing, which might be associated with loss of consciousness^10,23^. The second hypothesis suggests anesthesia to synchronize activity across large brain networks. This loss of spatial segregation might prevent structured information processing and thus lead to loss of consciousness^24–26^.

The result of stationary FC analysis reveals loss of connectivity between homotopic areas within the two hemispheres, which is in agreement with the first hypothesis. We verified our results in two separate studies using a slightly different experimental design: one group with individual animals measured at a defined isoflurane dose and a second group with dose-escalation in individual animals. We hence refrained from combining them. Both studies yielded the same results demonstrating the robustness of the findings, though we are aware that larger group sizes might have revealed additional statistically significant changes in brain networks in response to increasing anesthesia depth. This is also revealed by dynamic network analysis, which indicates reduced weight of dFSs that exhibit pronounced modular structure upon increasing the isoflurane dose. On the other hand, the contributions of dFS that lack modular structure across cortical and subcortical areas become dominant at an isoflurane dose of 2%. This is in line with the notion, that topological segregation is lost with increasing anesthesia depth, corresponding to the second hypothesis and in line with earlier studies in rats^36,44^ and mice^14^. Apparently, this widespread synchronization is averaged out when considering long integration intervals (stationary FC) and we observe loss of homotopic correlation as the prominent feature.

An interesting observation is that mice under all isoflurane levels displayed absence of cortico-thalamic stationary FC. While this is in line with reported absence of cortico-thalamic interaction during sedation^14,17,19,20,36,44,45^, it is in conflict with other reports describing diminished but detectable thalamo-cortical FC imposed by anaesthesia^15,16^. For example, Boveroux *et al*.^13^ found thalamo-cortical functional anti-correlations when studying propofol-induced unconsciousness in humans. Also in rats, anti-correlated FC between thalamus and cortex has been found^46^. These apparent discrepancies might indicate that the thalamo-cortical interaction is dependent on the type and depth of anesthesia. It should be emphasized that while we did not detect any significant cortico-thalamic interactions in stationary FC analysis of isoflurane anesthetized mice, we have identified dFSs that showed distinct cortico-thalamic interactions, which is in line with previous reports involving an isoflurane/medetomidne combination analysis in mice^8^. This indicates that these interactions are not fully suppressed by isoflurane but their contribution becomes too small to be detected when the integration interval is too long, i.e. a few minutes. This highlights the significance of complementary information that might be obtained from dynamic FC analysis.

Relating these observations to the isoflurane mechanism of action remains at this stage purely speculative. Decreased FC in cortical networks revealed by stationary FC analysis might reflect interference of isoflurane with glutamatergic neurotransmission, which will decrease the extent of synchronization of neurons connected via one or more synapses. This effect becomes more prominent upon increasing the isoflurane dose. This is also reflected by the fact, that dFSs showing cortical segregation become less prominent upon increasing the isoflurane dose due to reduced glutamate signaling resulting in reduced synchronization of spontaneous fluctuations of cortical neurons. The suppression of subcortical networks by isoflurane, as revealed by stationary FC analysis, might reflect GABA-ergic effects in view of the relatively high level of GABA receptors in the basal ganglia including the striatum. As already stated, these explanations are speculative and should be tested by using genetically engineered animals and/or pharmacological interventions targeting the various receptors suggested to be isoflurane targets.

An important aspect to be considered when analyzing the hemodynamic readouts constituting the fMRI signal is the stability of the physiological state of the subject. Physiological parameter recordings revealed constant oxygen saturation values throughout the experiment irrespective of the anesthetic doses used. This is to be expected as mice have been artificially ventilated at constant tidal volumes with a defined amount of oxygen provided via the respiration gas and allowed avoiding potential effects due to respiratory depression induced by anesthesia. Measurements of heart rate and pulse distention showed an overall decline with increasing dose of anesthesia indicative of reduced cardiac output. Since fMRI is an indirect measure of cerebral activation, which depends on the cerebrovascular baseline state, changes in blood pressure might affect the amplitude of the BOLD signal fluctuations and thereby potentially also the correlation analysis. This has also been reported previously in^47^ and may be considered a drawback of studying anesthesia-related effects using fMRI.

Apart from potential impact due to alteration in the physiological state, fMRI is hampered by its inherently low temporal resolution due to (i) the temporal characteristic of the hemodynamic response acting as strong temporal low-pass filter, and (ii) the sequential nature of MRI data sampling. Sampling rates are of the order of seconds, which puts a lower limit to a sampling interval in dynamic FC analysis of the order of 15 to 20 s, which is long compared to changes in microstates as reported on the basis of EEG measurements^7,29^. Changes occurring at sub-second time scales will be missed. Similarly the effect of the window length on dFS has also been widely discussed. Shakil *et al*.^48^ showed that clustering based on the sliding window correlation did not reliably reflect the underlying state transitions unless the window length was comparable to the state duration. However, here we have utilized the algorithm that uses dictionary-learning approach, which has been shown to reliably estimate the dFS with changing window lengths within a range of 15 to 50 s^7,29,49–51^. Additional aspects regarding dFC analysis relate to the proper number of atoms to be included and whether it is appropriate to keep the atoms fixed for group comparisons. Data reduction algorithms such as dictionary learning are vulnerable to over-fitting if too many components/atoms are selected. Indeed, it is possible to explain 100% of the variance if the number of component matches that of the number of frames. This would however fail to achieve the goal of reducing dimensionality. Many examples in the literature use 50–60% variance explained as a commonly used threshold choice. For instance previous work also based on dictionary learning has reported ~50% of variance explained for 16 atoms (Li *et al*., 2014), which is comparable to the results reported in this study. Similarly, Grandjean *et al*. could explain 50–60% of the variability using 20 atoms. In line with these reports and in order to avoid overfitting we restricted ourselves to 20 atoms. Nevertheless, the optimal number of atoms for dynamic FC analyses remains a point to be addressed in future work. Another point of consideration for future work would be to identify a method to perform statistical analysis without the constrain of keeping the atoms fixed across all groups, i.e. considering the potential appearance of new atoms in each group in the analysis.

Our results refer strictly to isoflurane as an anaesthetic and it is not obvious to what extent the findings regarding specific networks can be generalized to other anesthetic drugs, which have different modes of action and thus affect different brain networks^14^. Nevertheless, there may be some general principles: Grandjean *et al*.^14^ using stationary rs-fMRI demonstrated widespread cortical synchronization in mice exposed to high levels of urethane, i.e. loss of modular structure. This is analogous to loss of functional segregation observed under 2% of isoflurane and might indicate that is in fact an indicator of deep anesthesia, irrespective of the agent used.

In summary, we applied stationary and dynamic FC analysis to examine dose dependent effects of isoflurane anesthesia on functional networks in mice. We found that while stationary FC analysis with increasing dose of anesthetic revealed loss of functional connectivity between homotopic brain regions, dynamic FC analysis revealed loss of spatial segregation across some of the brain functional networks, which might reflect a state of deep anesthesia. Dynamic functional network analysis revealed significant interactions among functional networks that were not apparent from the conventional stationary analysis.

## Methods

### Animals, preparation, and anesthesia

The experiments were performed in compliance with Swiss laws on animal protection and approved by the Veterinary Office of the Canton of Zurich and in compliance with the ARRIVE guidelines. Female C57BL/6 mice (Janvier, Le Genest-St Isle, France) between 10 and 15 weeks old were studied. All mice were initially anesthetized with isoflurane in a 20% O_2_/80% air mixture: 3.5% for induction, 2% for endotracheal intubation and during set-up on the animal bed. Throughout the duration of the experiment, animals were mechanically ventilated using a small animal ventilator (CWE, Ardmore, USA) with a 20% O_2_/80% air mixture at a rate of 80 breaths/min, a respiration cycle of 25% inhalation, 75% exhalation, and an inspiration volume of 1.8 ml/min. The head was placed with the animal’s incisors secured over a bite bar and fixated by ear bars, ophthalmic ointment was applied to the eyes, and a rectal temperature probe was inserted to keep the animal at 36.5 ± 0.5 °C by means of a warm-water circuit integrated into the animal holder (Bruker Biospin GmbH, Ettlingen, Germany). The tail vein was cannulated for intravenous (i.v.) administration of the neuromuscular blocking agent pancuronium bromide (Sigma-Aldrich, Steinheim, Germany).

Two independent sets of studies were performed to evaluate the effects of isoflurane in a dose dependent manner.

Group 1 (Longitudinal design: dose-escalation in individual mice): Twelve animals were used in the experiment. Isoflurane (Abbott, Cham, Switzerland) was sequentially increased from 1.1% to 1.3%, 1.5% and 2.0% in a 20% O_2_/80% air mixture for each individual mouse. After each incremental increase of isoflurane concentration, there was a 10 min interval for equilibration before the fMRI data acquisition was started. Mice remained in the MR scanner throughout the duration of the experiment.

Group 2 (cross-sectional design: single dose for each mouse): Isoflurane was administered in a 20% O_2_/80% air mixture at a single defined dose per mouse in order to avoid any accumulation effects. Doses used were 1.1% (N = 10 mice), 1.2% (N = 19), and 1.5% (N = 18).

Each animal received an i.v. bolus injection of 0.5 mg/kg pancuronium bromide dissolved in saline (0.5 mg/3 ml) followed by a continuous infusion of 0.5 mg/kg/h of pancuronium bromide corresponding to an infusion rate of the solution of 3 ml/kg/h.

Animal preparation, anesthesia protocols, and the conditions during resting-state measurements were identical for the fMRI experiments and for the assessment of systemic physiological parameters. Approximately 20 min were used for animal preparation, and a further 20 min for preparatory MRI scans. Subsequently, rs-fMRI data sets of 6 min duration each were acquired. After the experiments, time for recovery from anesthesia and pancuronium bromide administration was provided for all the animals.

### fMRI

MRI/fMRI experiments were carried out using a Bruker Biospec 94/30 small animal MR system (Bruker BioSpin MRI, Ettlingen, Germany) operating at 400 MHz (9.4 T). A four-element receive-only cryogenic phased array coil (Bruker BioSpin AG, Faellanden, Switzerland) was used in combination with a linearly polarized room temperature volume resonator for transmission (Bruker BioSpin MRI, Ettlingen, Germany). Anatomical images acquired in the sagittal and horizontal direction allowed exact positioning of 12 adjacent coronal slices of 0.5 mm slice thickness, which were used for the rs-fMRI scans. A gradient-echo echo-planar imaging (GE-EPI) sequence has been used for rs-fMRI data acquisition with field of view = 16 × 7 mm^2^, matrix dimensions = 80 × 35, TR = 1 s, TE = 12 ms, flip angle = 60 degrees. The time series acquired was of 360 s length.

### Measurement of systemic physiological parameters

For physiological parameter measurement, the left hind limb of the mouse was shaved and a fiber optic pulse oximeter (MouseOx, STARR Life Science, Oakmont, USA) fixed to the flank in order to record heart rate (in beats per minute, bpm), pulse distention (in μm), and oxygen saturation (in %).

### Data Processing

All the pre-processing was performed using tools from FMRIB’s Software Library (FSL version 5). FSL’s recommended pre processing pipeline was used. Motion correction, removal of non-brain structures, high pass temporal filtering with sigma = 75.0 s; pre-whitening and global spatial smoothing using a 0.2 mm Gaussian kernel was applied to increase signal to noise ratio as part of the pre-processing.

After the pre-processing, the functional scans were aligned to the high-resolution anatomical AMBMC template (http://www.imaging.org.au/AMBMC/AMBMC) using linear affine and nonlinear diffeomorphic transformation registration as implemented in ANTs (ANTs. v 1.9; http://picsl.upenn.edu/ANTS/).

We used FSL’s MELODIC for probabilistic independent component analysis^52^. The multi-session temporal ICA concatenated approach, as recommended for rs-fMRI data analysis, allowed to input all subjects from all the groups in a temporally concatenated fashion for the ICA analysis. ConcatICA yielded different activations and artifact components without the need of specifying any explicit time series model.

A total of 100 independent components (IC maps) were extracted and the mixture model approach was applied on these estimated maps to perform inference analysis. An alternative hypothesis test based on fitting a Gaussian/gamma mixture model to the distribution of voxel intensities within spatial maps^53,54^ was used to threshold the IC maps. A threshold of 0.5 (p < 0.5) was selected for the alternative hypothesis in order to assign equal ‘cost’ to false-positives and false-negatives. Out of the 100 independent components (IC maps), only 25 numbers of components were selected, while the components that overlapped with vascular structures and ventricles were excluded from further analysis. Similarly, regions at the brain surface, which are prone to be affected by the motion artifacts due to e.g. breathing, were excluded.

Dual Regression (FSL 5.0.2.2) was used for between-subject analysis allowing for voxel-wise comparisons of rs-fMRI^55,56^. Dual regression is a technique that first regresses z-score group-IC maps (group spatial maps) into subject specific 4D resampled datasets to give a set of subject-specific variance normalized time courses for each component separately, and then regresses these time-courses into the same 4D dataset to get a subject-specific set of spatial maps. We used dual regression to generate the subject specific set of spatial maps from IC components.

Non-parametric permutation based inference^57^ was performed with the subject-specific component spatial maps concatenated across subjects for each analysis and submitted to voxel-wise between-subject analysis to test for effects of anesthetics dose dependency on functional connectivity using FSL-randomise^58^. Contrasts were set up using FSL’s general linear model (GLM) and 5000 randomized permutations were run as the FSL default setting. We used threshold-free cluster enhancement (TFCE)^59^ for statistical inference to validate the likelihood of extended areas of signal, which also takes into account information from neighboring voxels and combines the quality of both conventional cluster-based as well as voxel-based thresholding^59^. Correction for multiple comparisons across space was applied assuming an overall significance of *α* (p < 0.05) using permutation testing and TFCE. Bonferroni correction was applied separately to each analysis depending on the number of components of interest.

FSL Nets (FSLNets v0.6) was used for estimating the network model of rs-fMRI data. The partial correlation matrices of the BOLD signal time courses of each component from dual regression were then clustered to form a dendrogram. These clusters were used as input in to the GLM analysis and run through FSL-randomise^58^ to perform 5000 permutations to test for statistical significance. Edges, i.e. connections between network nodes showing statistically significant differences between the groups under consideration were obtained from GLM analysis. These significant network edges were then used to calculate the network box plots that take into account each edge and provide more information on difference in connectivity values between the groups. We applied FDR with multiple corrections method using the same unpaired t-test design matrix as used previously for dual regression analysis.

### Dynamic Functional Connectivity analysis

Dynamic functional connectivity networks were evaluated using the dictionary learning approach^6,7,29^. Time courses were extracted from the ICA maps and fed into the dFC algorithm. Simple building blocks (referred as ‘atoms’) of whole brain connectivity were estimated using dictionary learning algorithm^29^ with 30 folds each made of 200 iterations. Building blocks (atoms) were estimated for all anesthetic doses and all grouped together in a concatenated fashion, similar to ICA concatenation approach, and then were further regressed from the building blocks estimated from each anesthetic dose, similar to the dual regression approach. Atoms estimated were energy bounded. The algorithm was run iteratively many folds and matched to the first fold using Hungarian algorithm with spatial correlation as similarity measure in order to produce robust dictionary learning atoms or dynamic functional connectivity states (dFS). The patterns extracted from the algorithm explained more than 50% of the variance in the sliding window correlation matrix. The atoms generated by the algorithm are the transient states of functional connectivity and therefore it is important to perform statistical tests in order to find group differences. In order to compare the dFSs across different doses, we fixed their sequence to assure comparability across the study. A two-sample t-test was performed to statistically validate the results of the dynamic functional connectivity analysis.

## Electronic supplementary material

Supplementary material
