# Supplementary material for: Increasing isoflurane dose reduces homotopic correlation and functional segregation of brain networks in mice as revealed by resting-state fMRI
Source: Sci Rep. 2018 Jul 12;8:10591. doi: 10.1038/s41598-018-28766-3 (PMC6043584; doi:10.1038/s41598-018-28766-3)
Supplement: Supplementary file 1 — Supplementary material [file 41598_2018_28766_MOESM1_ESM.docx]

**Increasing isoflurane dose reduces homotopic correlation and functional segregation of brain networks in mice as revealed by resting-state fMRI**

**Bukhari Q^1^, Schroeter A^1^ and Rudin M^1, 2 *^**

^1^ Institute for Biomedical Engineering, ETH Zurich and University of Zurich, Zurich, Switzerland

^2^ Institute of Pharmacology and Toxicology, University of Zurich, Zurich, Switzerland

**Correspondence:**

rudin@biomed.ee.ethz.ch

Prof. Markus Rudin, PhD

Institute for Biomedical Engineering

University of Zürich and ETH Zürich

HIT E22.4

Wolfgang-Pauli-Strasse 217

CH-8093 Zürich, Switzerland

# Supplementary Material

**Supplementary Table 1**: P-values for pairwise comparisons for changes in FC strengths upon increasing the isoflurane dose for ICs belonging to lateral cortical and associative cortical networks (Limb cortex: S1; motor cortex: M1; somatosensory S1 (see Fig. 3a, main text)

| Isoflurane dose | Limb Cortex  left vs right | Motor cortex M1  left vs right | Somatosensory S1  left vs right |
| --- | --- | --- | --- |
| **1.1 vs 1.3** | .09 | .002 | .028 |
| **1.1 vs 1.5** | .10 | .07 | .08 |
| **1.1 vs 2.0** | .001 | .001 | .001 |
| **1.3 vs 1.5** | .10 | .11 | .11 |
| **1.3 vs 2.0** | .08 | .001 | .002 |
| **1.5 vs 2.0** | .07 | .001 | .001 |

**Supplementary Table 2: One-way Anova to identify a dependence of FC network on isoflurane dose.** Null hypothesis is that there is no dosage effect. The null hypothesis (no effect of varying isoflurane dose) is rejected at a p-value of 1.39⋅10^-9^.

| **Source** | **SS** | **df** | **MS** | **F** | **Prob>F** |
| --- | --- | --- | --- | --- | --- |
| **Columns** | 138.2094061 | 3 | 46.06980202 | 24.94171735 | 1.3910^-9^ |
| **Error** | 81.27232222 | 44 | 1.847098232 | - | - |
| **Total** | 219.4817283 | 47 | - | - | - |
|  |  |  |  |  |  |

**Supplementary Table 3:** **P-values indicating significant change in weight of the dynamic functional states upon increasing the isoflurane dose from 1.1 to 2.0%** (Fig. 6, main text)

| Dynamic functional  state (atom) | Isoflurane dose  1.1% vs. 2.0% |
| --- | --- |
| **dFS # 2** | .009 |
| **dFS # 6** | .001 |
| **dFS # 9** | .001 |
| **dFS # 10** | .001 |
| **dFS # 14** | .008 |
| **dFS # 15** | .005 |

**
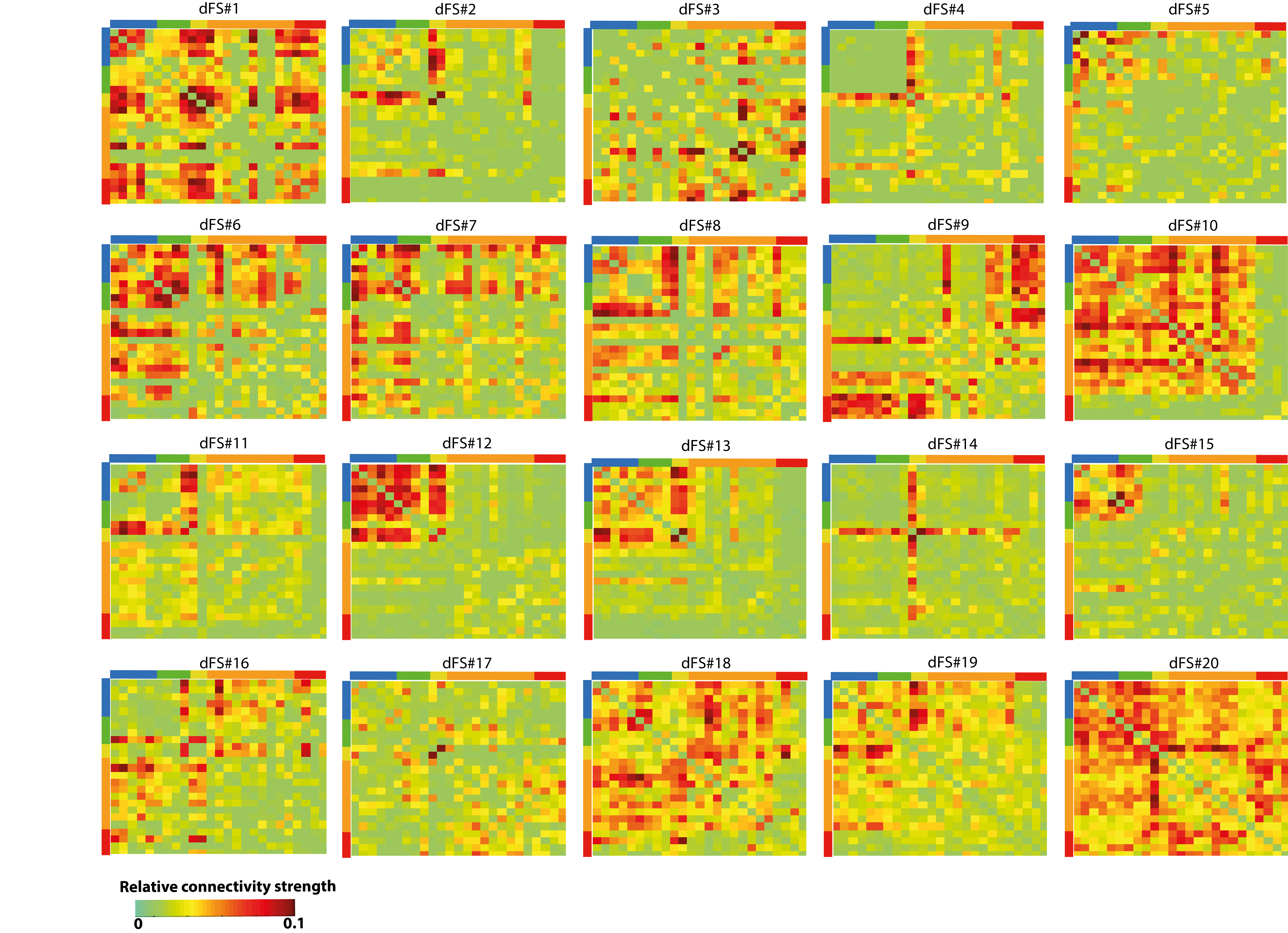
Supplementary Figures**

**Supplementary Figure 1: Twenty dynamic function states (dFSs, atoms) derived from dictionary learning dFC analysis.** The majority of dFSs presents a high degree of (modular) structure and revealed network interactions that were masked in the stationary FC analysis. Modules are colour coded as (column at left and row at top of matrices): LCN = blue, ACN = green, PFN= yellow, SuCN = orange, thalamus = red.


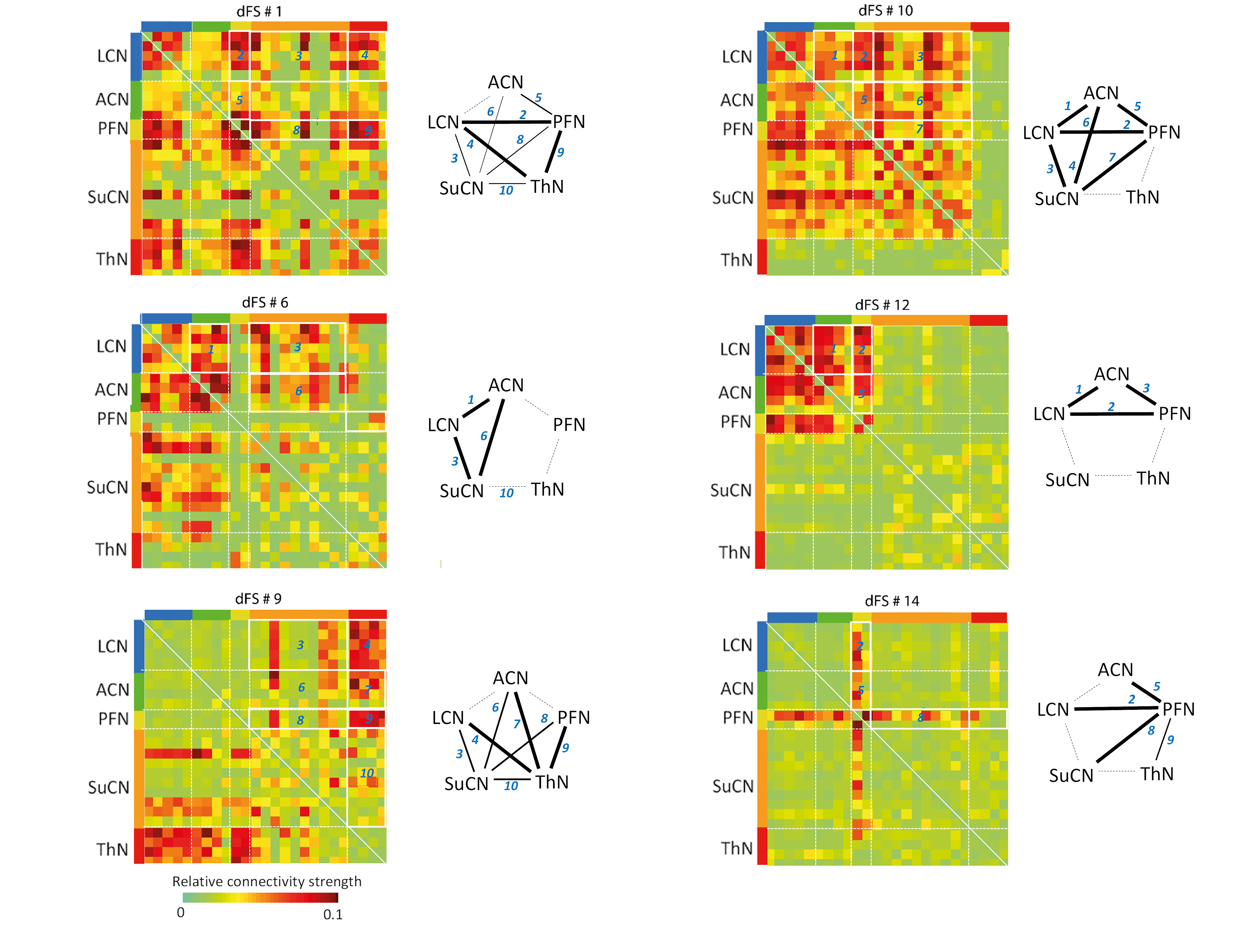


**Supplementary Figure 2: Selected dFS of mouse brain obtained by concatenating data from all anaesthetic doses tested.** Atoms (dFSs) generated from the dictionary learning dFC analysis show structure related to functional modules. White frames indicate dominant in-between network interactions. These interactions are illustrated in the network graph representing the network consisting of five modules. Modules are colour coded as (column at left and row at top of matrices): LCN = blue, ACN = green, DMN = yellow, SuCN = orange, ThN = red.

**Cortico-thalamic FC: Analysing the mean amplitude values of thalamus and cortex**

**Introduction**

The connectivity between thalamus and cortex was found to be absent irrespective of the isoflurane dose and in line with earlier reports. As connectivity is inferred from the correlation of spontaneous BOLD fluctuation in to regions-of-interest, low z-scores may reflect 1) a weak correlation of the BOLD signals or 2) reflect lack of sensitivity in detecting correlations due to low signal-to-noise ratio in either or both of the regions involved. The second option has to be considered as we have used a surface coil for signal detection, i.e. sensitivity decreases with increasing distance from the coil.

In order to determine the basis of low cortico-thalamic FC under isoflurane, we analyzed the mean amplitude values of BOLD fluctuations in thalamus and cortex at varying levels of anesthesia


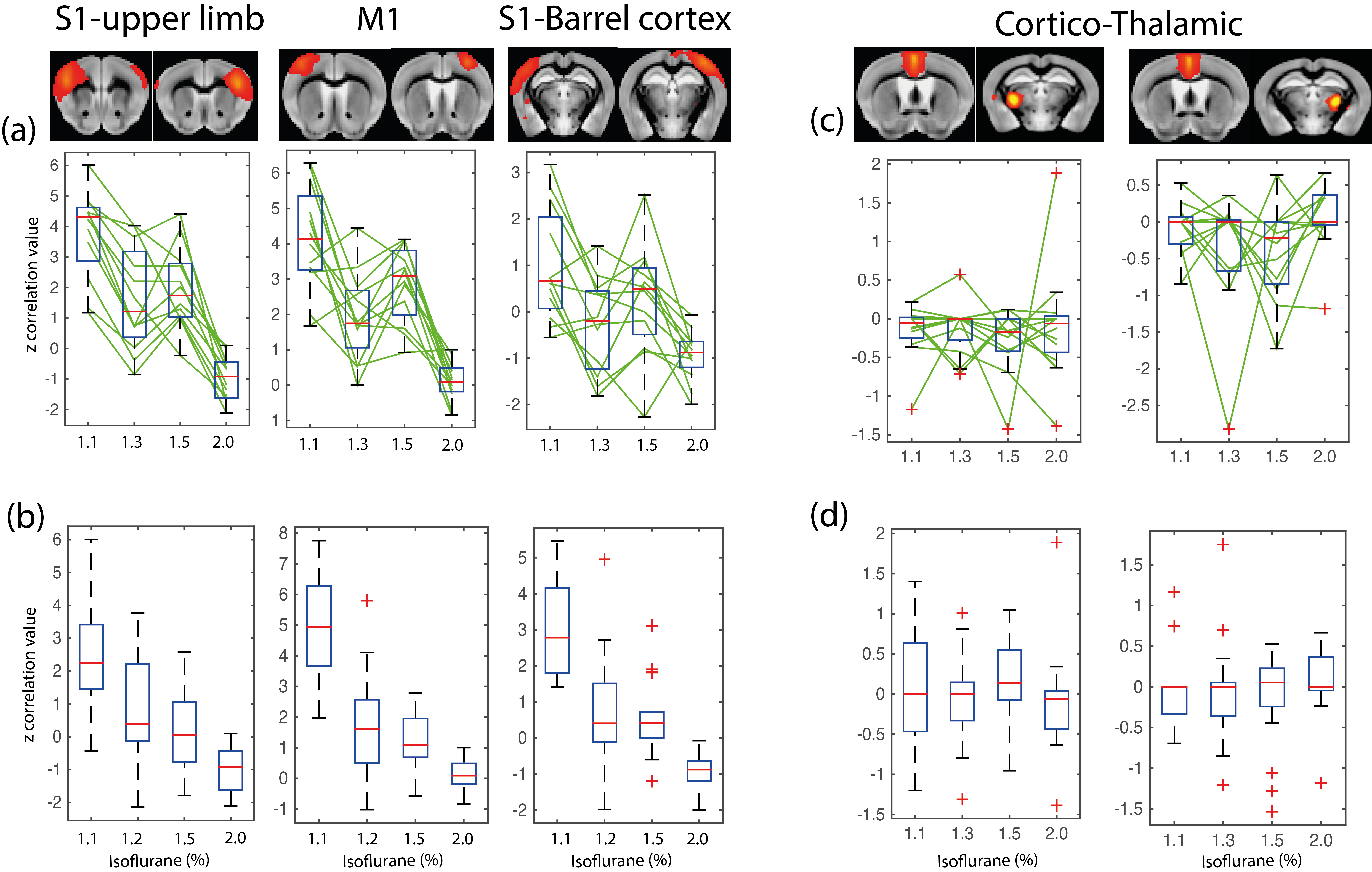


**Supplementary Figure 4**: **Cortical-thalamic FC as a function of isoflurane level.** Regions of interest in PFC/Cg and vTh (upper panels) and FC (z-score) for cortio- thalamic FC for left and right vTH, respectively.

**Methods**

Signal amplitudes were analyzed in a cortical region close to the coil (PFC/Cg; Suppl. Fig. 4) and in the ventral thalamic region (vTh; Suppl. Fig.4). Mean signal amplitudes and standard deviations were then extracted from the time series associated with the corresponding independent component. All the time series were mean centered.

**Results**

A representative BOLD signal trace for left ventral thalamus (vTh) and PFC/Cg at an isoflurane dose of 2% are shown in Suppl. Fig. 5a, 5b, respectively. As expected, SNR is higher in cortical and subcortical regions of interest. Also, PFC BOLD trace displays characteristic peaks, which might be related to cortical slow wave activity (Schwalm M, et al. (2017) ELife 6: e27602), though further studies combining calcium recordings with fMRI would be required to address this in more depth. Results of the quantitative analysis are given in Table 1.


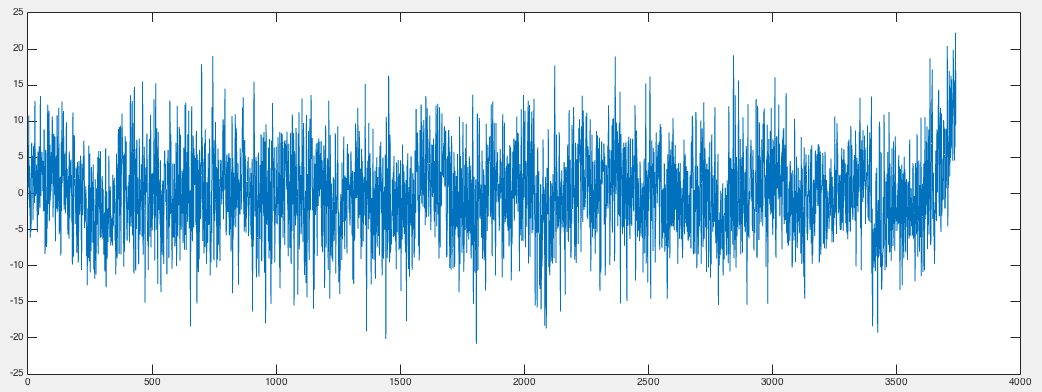
a)


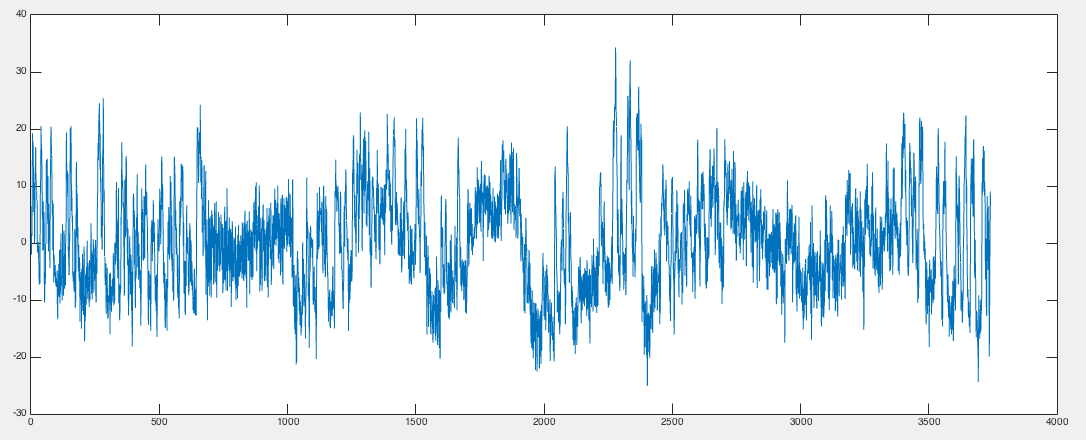


b)

**Supplementary Figure 5**: BOLD time course for left vTh (a) and PFC/Cg (b) for the ICs depicted in in Fig.1.

**Supplementary Table 4**: Mean amplitude and standard deviation of BOLD signal trace for regions-of-interest in left and right vTh and PFC/Cg,

| Region | Isoflurane (%) | 1.1 | 1.3 | 1.5 | 2.0 |
| --- | --- | --- | --- | --- | --- |
| Left vTh | mean | -7.18e-05 | -6.19e-04 | -0.0017 | 2.23e-04 |
|  | Std | 6.16 | 5.76 | 5.95 | 5.81 |
| Right vTH | mean | 1.99e-04 | 9.18e-04 | -8.33e-04 | -0.0016 |
|  | Std | 6.30 | 5.64 | 5.76 | 5.24 |
| PFC/Cg | mean | 0.0021 | 7.72e-04 | -6.49e-04 | 1.96e-04 |
|  | Std | 14.03 | 5.19 | 9.06 | 8.67 |

**Discussion**

While the standard deviation as measure of the amplitude of BOLD fluctuation are not very different for the two regions, the PFC/Cg display more features (spikes), which are not observed for thalamus either due to inferior sensitivity or due to the fact that there are no such spikes in the thalamic signal. An interesting finding is that dynamic FC analysis reveals involvement of the thalamus, i.e. 4 out of 20 atoms analyzed reflect interactions with the thalamic ICs (Fig 5,6; Suppl. Fig 1,2). This indicates that the absence of cortico-thalamic FC in stationary FC analysis is not primarily a sensitivity issue, but might be due to the fact that the contribution of dFSs displaying thalamic involvement to the overall stationary signal is too small to be detected.

**Potential interference of physiological parameters pulse distention and heart rate with BOLD fMRI readouts**

**Introduction**

As the BOLD signal is of hemodynamic nature it is conceivable that any changes in cardiovascular parameters such as heart rate and blood pressure might affect the results. We therefore analyzed whether there is any group effect of stationary or dynamic FC results caused by changes in pulse distention (as measure of blood pressure) or heart rate.

**Methods**

In order to identify any significant group effect of pulse distention or heart rate on FCs, we multiplied the ICs of each group with a coefficient $ci_{i}$ that corresponded to the differences of the physiological parameter $p_{i}$ from the mean value across all groups, i.e.

$$c_{i}=p_{i}-\frac{1}{n}\sum_{j=1}^{n} p_{j} , n=4$$

and correspondingly

${IC}_{j,i}'=IC_{j}\cdot c_{i}$ with $j=1,\ldots,25$ and $i=1,\ldots,4 .$

${IC}_{j,i}'$ were then used to ran randomized permutation to check for significance. We used the dual regression dr2*.* files, which were the group-concatenated maps per IC. We divided each of these IC files in to group wise pairs

*Group 1 = isoflurane dose 1.1%*

*Group 2 = isoflurane dose 1.3%*

*Group 3 = isoflurane dose 1.5%*

*Group 4 = isoflurane dose 2.0%*

*IC1 🡪 Group 1, Group 2, Group 3, Group 4*

*.*

*.*

*IC100 🡪 Group 1, Group 2, Group 3, Group 4*

Here, group refers to the isoflurane dose and ICs refer to the 25 IC components that we had selected.

**Results**

The results of pulse distention and heart rate effect are given in Supplementary Table 5 and 6.

**Supplementary Table 5:** Pulse Distention per group and coefficient $c_{i}\left( PD \right)$of design matrix

| Group | 1 | 2 | 3 | 4 |
| --- | --- | --- | --- | --- |
| Isoflurane (%) | 1.1 | 1.3 | 1.5 | 2 |
| PD (μm) | 17 | 15 | 12 | 6.2 |
| Design matrix $c_{i}(PD)$ | 5 | 3 | -1 | -7 |

As the design matrix comprises positive and negative values the resulting in

*Positive effect: Group 1 x 5 + Group 2 x 3 🡪 IC1 . . . IC25*

*Negative effect: Group 3 x -1 + Group 4 x -7 🡪 IC1 . . . IC25*

In a second step, we used a design matrix (1,-1) to account for positive or negative values and tested for significant differences between the two conditions using randomized permutation. We ran 5000 randomized permutations but did not find any significant effect in any of the ICs. In conclusion, we did not find a significant effect of pulse distention on ICs and correspondingly FCs in the groups.

The same procedure was repeated for the heart rate (Supplementary Table 6). Again, we ran 5000 randomized permutations to determine the significance, but did not find any significant effect of heart rate on ICs and correspondingly FCs in the groups.

**Supplementary Table 6:** Mean heart rate per group and coefficient $c_{i}\left( HR \right)$of design matrix

| Group | 1 | 2 | 3 | 4 |
| --- | --- | --- | --- | --- |
| Isoflurane (%) | 1.1 | 1.3 | 1.5 | 2 |
| HR (beats per minute) | 560 | 540 | 530 | 500 |
| Design matrix $c_{i}(HR)$ | 16 | 4 | 1 | -21 |

**Conclusion**

We did not find a statistical significant interaction between the effects of the anesthetic on cardiovascular physiological parameters with those on the FC. However, absence of statistical significance does not imply absence of an effect. For example, it is conceivable that if the functional connectivity is actually causing an inverse (1/physio) weight to the FC values derived from BOLD signal fluctuations, then the method used would just normalize the physiology imposed artifact and no difference would be found.
